# Three-dimensional automated segmentation of adolescent idiopathic scoliosis on computed tomography driven by deep learning: A retrospective study

**DOI:** 10.1097/MD.0000000000042644

**Published:** 2025-05-30

**Authors:** Yong Ji, Xiajin Mei, Rong Tan, Wenxin Zhang, Yuliang Ma, Yun Peng, Yingchun Zhang

**Affiliations:** a Department of Spine II, The Ninth Medical Center, Chinese PLA General Hospital, Beijing, China; b College of Automation, Hangzhou Dianzi University, Hangzhou, Zhejiang, China; c Department of Biomedical Engineering, University of Houston, Houston, TX.

**Keywords:** computer-assisted, convolutional neural networks, deep learning, image processing, scoliosis, spine

## Abstract

Accurate vertebrae segmentation is crucial for modern surgical technologies, and deep learning networks provide valuable tools for this task. This study explores the application of advanced deep learning-based methods for segmenting vertebrae in computed tomography (CT) images of adolescent idiopathic scoliosis (AIS) patients. In this study, we collected a dataset of 31 samples from AIS patients, covering a wide range of spinal regions from cervical to lumbar vertebrae. High-resolution CT images were obtained for each sample, forming the basis of our segmentation analysis. We utilized 2 popular neural networks, U-Net and Attention U-Net, to segment the vertebrae in these CT images. Segmentation performance was rigorously evaluated using 2 key metrics: the Dice Coefficient Score to measure overlap between segmented and ground truth regions, and the Hausdorff distance (HD) to assess boundary dissimilarity. Both networks performed well, with U-Net achieving an average Dice coefficient of 92.2 ± 2.4% and an HD of 9.80 ± 1.34 mm. Attention U-Net showed similar results, with a Dice coefficient of 92.3 ± 2.9% and an HD of 8.67 ± 3.38 mm. When applied to the challenging anatomy of AIS, our findings align with literature results from advanced 3D U-Nets on healthy spines. Although no significant overall difference was observed between the 2 networks (*P* > .05), Attention U-Net exhibited an improved Dice coefficient (91.5 ± 0.0% vs 88.8 ± 0.1%, *P* = .151) and a significantly better HD (9.04 ± 4.51 vs. 13.60 ± 2.26 mm, *P* = .027) in critical scoliosis sites (mid-thoracic region), suggesting enhanced suitability for complex anatomy. Our study indicates that U-Net neural networks are feasible and effective for automated vertebrae segmentation in AIS patients using clinical 3D CT images. Attention U-Net demonstrated improved performance in thoracic levels, which are primary sites of scoliosis and may be more suitable for challenging anatomical regions.

## 1. Introduction

In spine surgery, enabling technologies, such as robotic surgery, augmented reality, and surgical navigation, have demonstrated great values in various surgical tasks.^[1–3]^ Central to these enabling technologies is the automatic 3-dimensional (3D) vertebrae segmentation using preoperative or intraoperative computed tomography (CT) images. For example, in pedicle screws trajectory planning^[4–6]^ or real-time surgical navigation,^[7–9]^ a reliable 3D vertebrae segmentation ensures the accurate reconstruction of important vertebral structures that can minimize complications associated with suboptimally placed pedicle screws or interbody cages.

Over the past decade, with the phenomenal development of artificial intelligence, deep learning-based vertebrae segmentation has achieved great success.^[10]^ A recent systematic review by Qu et al identified a total of 24 studies published between 2015 and 2021.^[11]^ Their review revealed that the U-Net neural network, a popular deep learning tool to identify specific structures within medical images, has been predominantly employed in vertebrae segmentation.^[12]^ The accuracy is comparable to manual segmentation results by trained practitioners.

Despite notable progresses, vertebrae segmentation of patients with adolescent idiopathic scoliosis (AIS) remains a significant challenge, due to the complicated 3D geometric deformities in the spinal curvature and vertebral shape. Variations in spinal anatomy across patients have been known difficulties in spinal vertebral segmentation (VerSe).^[13]^ For AIS patients, the problem is more difficult because of the deformity in all 3 anatomical planes, asymmetry in single vertebrae, different classifications and variable severity.^[14]^ Few studies explored 3D vertebrae segmentation of AIS spines.^[12,15]^ Antico et al employed a convolutional neural network (CNN) model to segment thoracic spines (T5–T12) using MR images from a sample of 25 AIS patients.^[16]^ Bennström and Winzell developed a U-Net-based segmentation model trained on a heterogeneous set of CT data of AIS spines.^[17]^ Meanwhile, the majority of reported deep learning studies of AIS focused on applications such as scoliosis classification,^[18]^ segmentation on 2D radiographs,^[18]^ and Cobb angle measurement.^[19–21]^ The findings from these research studies indicate that segmenting spines in AIS cases continues to be a complex challenge and remains an area that has not been thoroughly investigated. This is mainly due to the fact that CT image data of spines in AIS cases are difficult to obtain, and the existing segmentation algorithms are not robust enough and have complicated segmentation procedures when dealing with spines with complex anatomical structures.

To address the knowledge gap in the field, this study leverages high-quality clinical CT images sourced from a high-volume hospital and aims to meet the escalating demand for reliable vertebrae segmentation methods for AIS spines, a demand driven by the proliferation of enabling technologies in AIS treatment.^[22]^ The specific objective is to develop and validate advanced deep learning methods to provide accurate vertebrae segmentation. Segmenting vertebrae in CT images of adolescents with idiopathic scoliosis and conducting quantitative measurements and analyses based on medical imaging indicators before and after treatment can help doctors develop treatment plans for patients. Two popular networks, namely the U-Net and the Attention U-Net, are evaluated in the task of AIS spine segmentation. U-Net is a type of CNN architecture particularly suited for biomedical image segmentation tasks. The U-Net architecture is essentially an encoder (downsampling) path followed by a decoder (upsampling) path, which together give it a “U” shape. The encoder part of U-Net captures the context in the image, while the decoder uses this information to enable precise localization. Attention U-Net, on the other hand, is a variant of U-Net that incorporates an attention mechanism into the U-Net architecture, and has been also used in medical image segmentation.^[23]^ The attention mechanism helps the network to focus on certain parts of the image over others, depending on where the network “believes” the most important information is located; therefore, Attention U-Net has the chance to further improve the segmentation performance particularly for difficulty regions. We hypothesized that both networks could achieve satisfactory segmentation results for AIS spines, and that the Attention U-Net may show better performance at the main site of scoliosis.

## 2. Materials and methods

### 2.1. Study design

This study retrospectively evaluated the efficacy of deep learning models in VerSe from CT images of AIS patients. The primary objective was to assess the feasibility and effectiveness of U-Net and Attention U-Net networks in providing accurate VerSe in AIS patient CT images. Our initial data compilation comprised 41 AIS patients admitted to our hospital between 2018 and 2022. The final dataset was narrowed down to 31 AIS patients with complete spine CT images, ranging from cervical to lumbar, after excluding 10 patients due to incomplete CT data. Within this cohort, males constituted 16.1% with a total of 5 patients. The average age of the cohort was 14.7 ± 3.4 years, with a mean height of 1.60 ± 0.06 m, and an average BMI of 18.5 ± 2.9 kg/m^2^. The distribution of the Lenke types was as follows: 17 were classified as Lenke I, 4 as Lenke II, 3 as Lenke III, 5 as Lenke V, and 2 as Lenke VI. The group demonstrated an average main Cobb angle of 52.1 ± 11.5°, indicating a substantial curve in the spine. The ground truth segmentation was semiautomatically annotated by experienced annotators using 3D Slicer (National Institutes of Health, Bethesda) and verified by experienced spine surgeons. The study was approved by the Medical Ethics Committee of the PLA Strategic Support Force Medical Center (approval no.: 2022-12; July 2022).

### 2.2. Training conditions

All networks were trained on servers equipped with RTX3090 GPU, utilizing the SGD optimizer for training optimization. The batch size was set to 3, the initial learning rate was 0.001, and the training epoch was 50.

### 2.3. Experiment tools

All experiments were conducted using PyCharm version 2022 (JetBrains, Czech Republic) with Python version 3.9.

### 2.4. Deep learning models

Numerous deep learning approaches have been utilized for the segmentation of 3D spine CT images. As per recent literature, the U-Net framework and its derivatives are most prevalently implemented in spine segmentation applications. U-Net, a highly advanced CNN structure, is typically utilized in biomedical image segmentation tasks. The “U”-shaped architecture of U-Net consists of a contraction (downsampling) pathway and an expansion (upsampling) pathway. The contraction pathway captures contextual information via convolutions and max pooling, while the expansion pathway ensures precise localization through the use of transposed convolutions. In addition, the structure integrates skip connections to merge low-level feature maps with high-level ones, thereby conserving critical detail.

In this research, we employed the fundamental U-Net architecture and its derivative, the Attention U-Net.^[12]^ The Attention U-Net incorporates an attention mechanism into the traditional U-Net structure, enabling the model to concentrate on specific regions within the image. This architecture effectively addresses the problem of class imbalance prevalent in segmentation tasks by directing the network’s attention towards certain target regions. This attention gating mechanism selectively accentuates relevant features while suppressing irrelevant ones, enhancing the model’s performance, especially in intricate or challenging segmentation tasks.

### 2.5. Workflow and performance metrics

**Figure 1.**
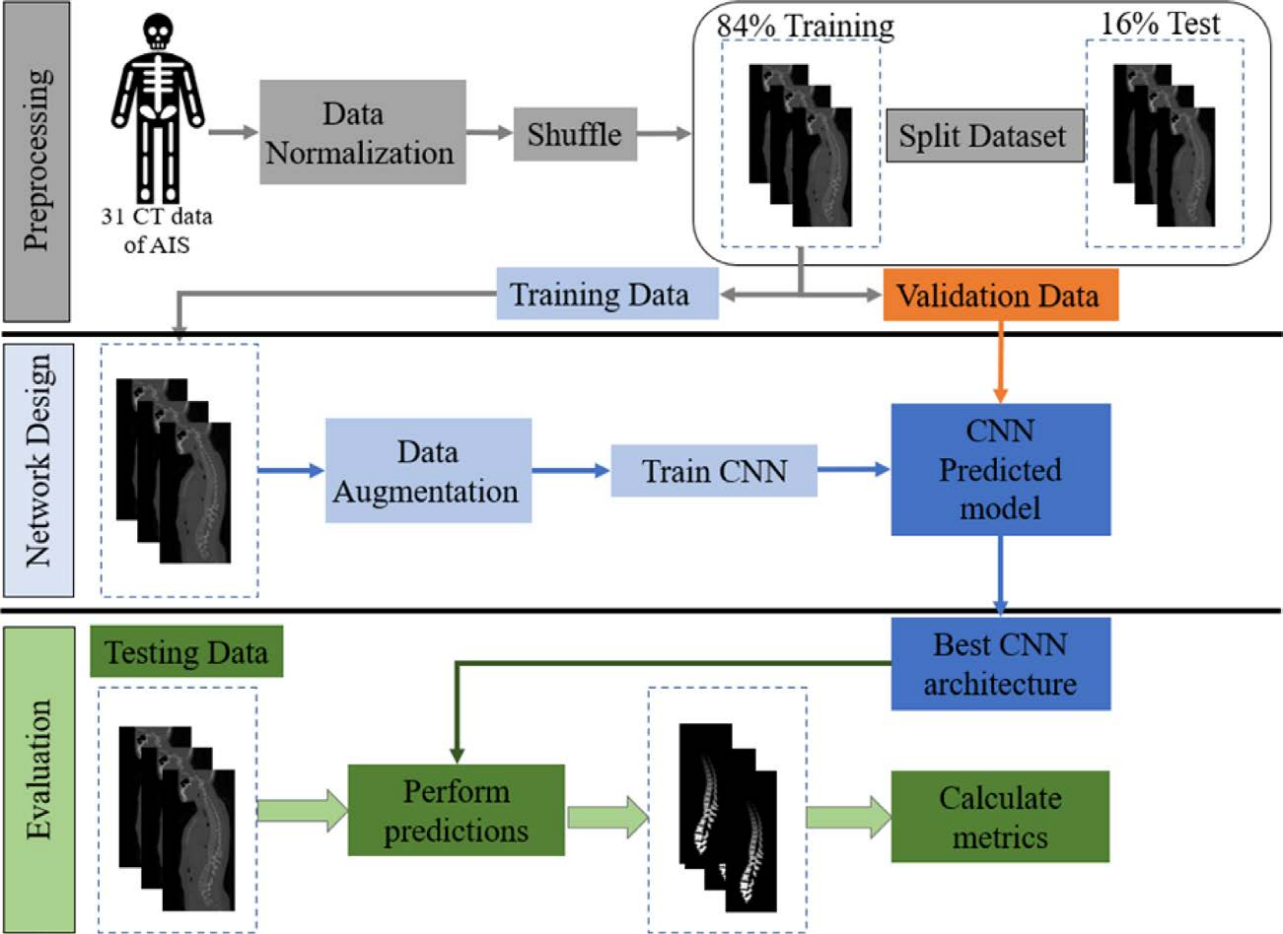
Workflow of the study. AIS = adolescent idiopathic scoliosis, CNN =convolutional neural network, CT = computed tomography.

The methodological framework for this study is depicted in Figure 1. The initial preprocessing step involved normalization and randomization of the CT data. Of the total 31 CT images, 21 were allocated for training, 5 for validation, and the remaining 5 for testing. Subsequently, data augmentation techniques including scaling and rotation were applied, resulting in a total of 5040 data points for model training. The augmented training dataset was then inputted into the selected neural networks (U-Net and Attention U-Net).

Throughout the training phase, the parameters of the neural network were iteratively optimized, retaining the most effective network architecture based on the outcomes from the validation set. This strategy was aimed at averting overfitting and ensuring the generalizability of the optimized network. Following this, the trained networks were evaluated using the test set for final performance appraisal, utilizing established performance metrics^[18,24]^:

• Dice Coefficient Score, which evaluates the overlap of pixels between automatic and ground truth segmentations.
• Hausdoff distance, which quantifies the maximum discrepancy between 2 sets in a metric space.

### 2.6. Statistical analysis

Comparative analysis of the performance metrics between the 2 deep learning models (baseline U-Net and Attention U-Net) was conducted using paired *t*-tests, based on data from identical patients in the test set. The significant level was set at 0.05.

## 3. Results

**Figure 2.**
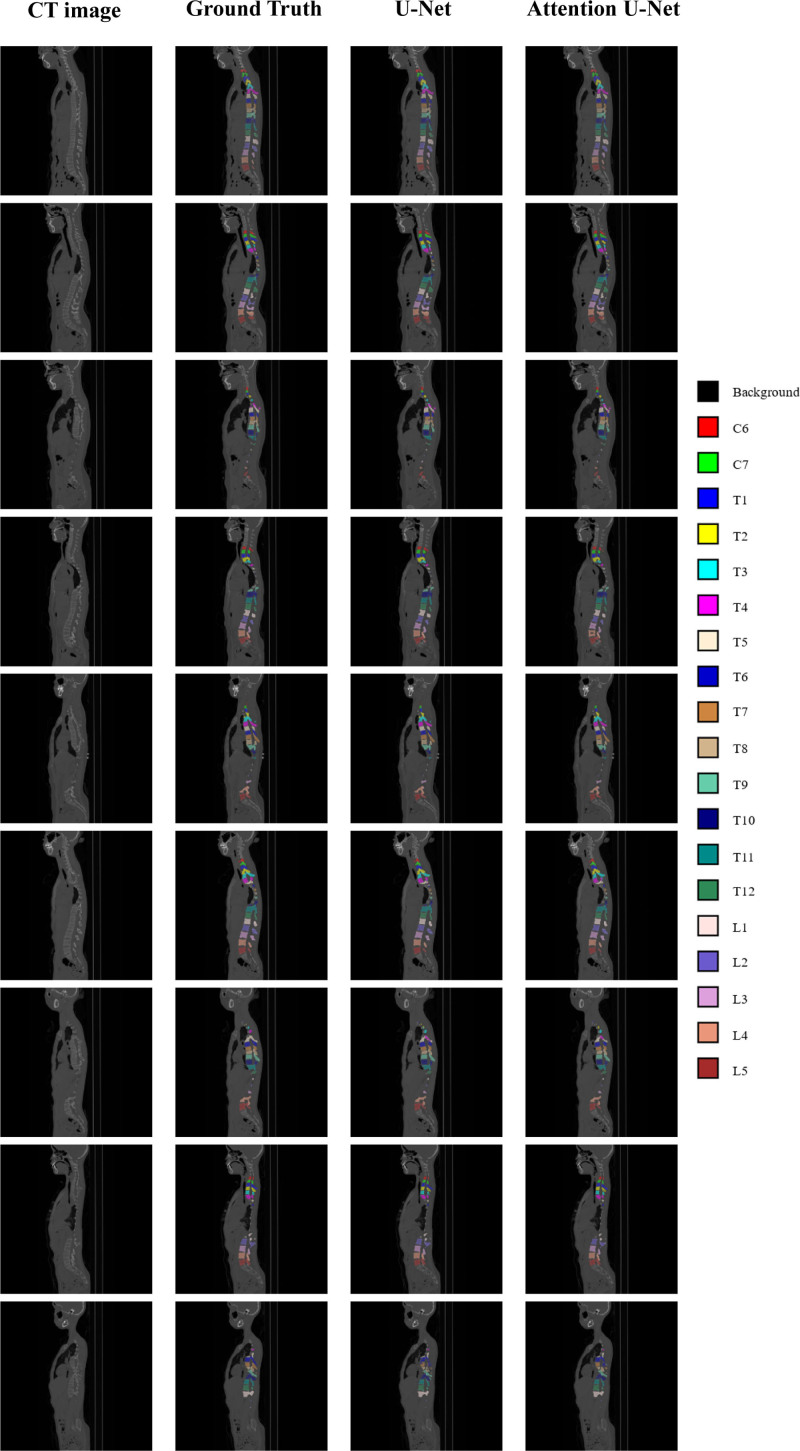
Representative CT images from the test group (from left to right: raw CT images, ground truth annotation, results by 3D U-Net and results by 3D attention U-Net). CT = computed tomography.

**Figure 3.**
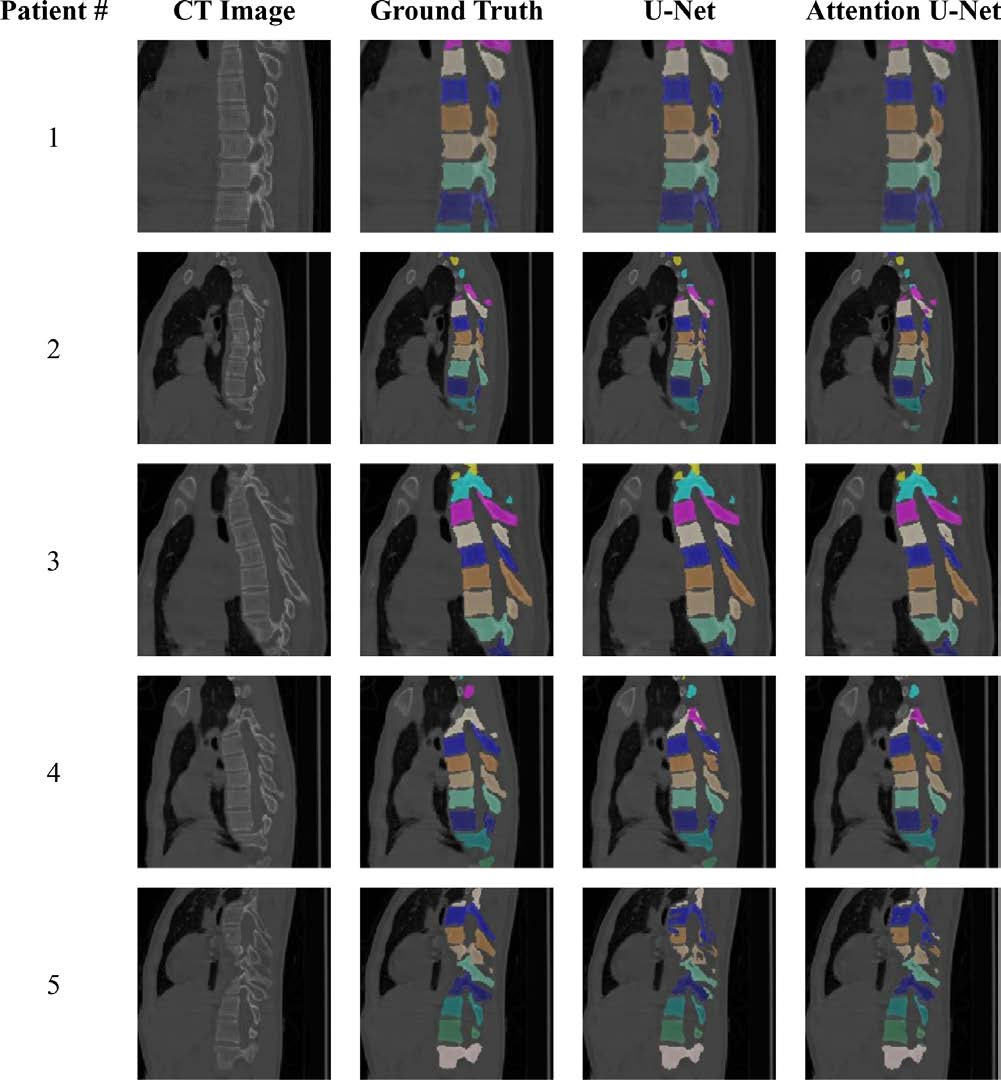
Representative images (zoomed in) of segmentation results at the thoracic region. Incorrect segmentation by the U-Net was shown in red rectangles. CT = computed tomography.

Both U-Net and Attention U-Net successfully identified all vertebral levels from C6 to L5 for all patients in the testing group (Fig. 2). However, upon detailed check, instances of incorrect labeling were discernible in the outputs from U-Net, particularly at levels exhibiting significant deformity (Fig. 3). These inconsistencies were not observed in the results produced by Attention U-Net.

Statistical analysis was performed using Python SciPy. The Dice coefficient scores and Hausdorff distances were 1st evaluated for normality using the Shapiro–Wilk test due to the relatively small sample size. A *P*-value > .05 from the Shapiro–Wilk test indicated that data did not significantly deviate from a normal distribution, confirming suitability for parametric testing. Data were then summarized as mean ± standard deviation. Table 1 reports the average Dice Coefficient Score and Hausdoff distance for all patients, with each patient’s results representing the average across all vertebral levels from C6 to L5. U-Net achieved an average Dice Coefficient Score of 92.2 ± 2.4% and Hausdoff distance of 9.80 ± 1.34 mm. Attention U-Net yielded similar outcomes, with a Dice Coefficient Score of 92.3 ± 2.9% and Hausdoff distance of 8.67 ± 3.38 mm. Given the paired design of this study – each patient’s anatomy was evaluated by both U-Net and Attention U-Net – a paired-sample *t*-test was chosen to evaluate differences between the 2 methods. The equality of variances was assessed using Levene’s test to ensure assumptions for the paired *t*-test were met. The paired *t*-test showed no statistically significant difference between the 2 network performances (*P* > .05); however, in the main sites of scoliosis (mid-thoracic), the Attention U-Net demonstrated improved Dice Coefficient Score (91.5 ± 0.0% vs 88.8 ± 0.1%, *P* = .151) and significantly improved Hausdoff distance (9.04 ± 4.51 mm vs 13.60 ± 2.26 mm, *P* = .027), suggesting a better suitability for complex anatomy.

**Table 1:** Summary of results and review of literature data.

Reference	Network type	Image type (CT only)	DCS (%)	HD (mm)
Current study*	U-Net	AIS (C6-L5)	92.2 ± 2.4	9.80 ± 1.34
Current study*	Attention U-Net	AIS (C6-L5)	92.3 ± 2.9	8.67 ± 3.38
Meng et al^[19]^	e.g., TRANS3DUNET	Normal (C6-L5)	85.5–91.1	6.69–8.39
Tao et al^[25]^	Transformer	Normal (C7-L5)	90.1 ± 0.94	6.68 ± 4.12
Wu et al^[26]^	*LVLS-HVPFE*	Normal (L1-L5)	95.7 ± 0.3	–
Sekuboyina et al†^,[18]^	Multiple	Normal (C6-L5)	83.9–91.7	5.80–9.57
Qu et al‡,^[11]^	Multiple	Normal	82.3–97.1	–

CT = computed tomography, DCS = Dice Coefficient Score; HD = Hausdorff distance.

* Paired *t*-tests were performed to detect difference in the DCS and the Hausdoff distance between U-Net and Attention U-Net, with no significance identified.

† This report reviewed results of 26 studies that participated in the VerSe 2019 and 2020 challenge (before 2021). Results shown here are the range of the top 5 studies.

‡ This review included 24 studies between 2015 and 2021 that reported segmentation results. Results shown here are the range of 8 studies that were based on CT spine images.

## 4. Discussion

Our study represents a preliminary evaluation of the potential of deep learning-based vertebrae segmentation for AIS spines using CT images. A crucial prerequisite for successful surgical navigation and robotic surgery is the precise segmentation of vertebral levels. Despite the growing demand for such technologies, there are scant published results on the segmentation of AIS spines. Our results demonstrated a satisfactory segmentation performance of the Attention U-Net network, both in normal and deformed vertebral levels.

Automated 3D segmentation of CT data on normal spines has been the subject of numerous studies.^[10,11,19–21,25,27,28]^ Even when applied to the more challenging AIS anatomy, our study’s Dice coefficient (92.2%) and Hausdoff distance (8.67 mm) results fall within the range of published data on normal spines (Dice coefficient 83.9–91.7%; Hausdoff distance 5.8–9.57 mm), according to a recent summary report of 26 studies participating in the VerSe challenges, and more recent state-of-the-art studies employing complex deep learning algorithms (Dice coefficient from 90.1% to 95.7%).^[23,25,26]^ The encouraging results substantiate the feasibility of using deep learning methods for the automated 3D segmentation of AIS spines. The satisfactory performance in our study could be attributed to the robustness of the modern U-Net segmentation network, the effective data augmentation method used in our study, and the high-quality of CT images.

Deep learning applications in the field of AIS surgery have mainly been focused on tasks such as automated scoliosis detection and classification,^[29]^ vertebrae localization and segmentation on 2D radiographs^[29]^ and Cobb angle measurement,^[30–32]^ with very limited data available on segmentation tasks. Our review of this field identified only 2 studies that reported segmentation results from AIS spine imaging data. In one of these studies, Antico et al (2021) employed a CNN to segment 2D image patches derived from MR images, specifically focusing on the T5 to T12 vertebral levels from a cohort of 25 AIS patients. They reported Dice coefficients in the range of 88% to 90%. In another study, a thesis by Bennström and Winzell, the authors utilized a 3D voting algorithm on CT spine images obtained from 19 subjects, reporting a Dice coefficient of 92.7 ± 0.02%. Direct comparison of these results with our data is confounded by the differences in image type (2D image, MR images) and the restricted anatomical region under examination (T5–T10). With the increasing demand for surgical enabling technologies such as robotic-assisted pedicle screw insertion and surgical navigations for AIS patients, there is an unmet need for more accurate segmentation tools for the AIS surgeries. Our study adds to the literature by demonstrating the feasibility and characterizing the performance results of the commonly used 3D medical image segmentation algorithm, 3D U-Net and its variant, Attention U-Net, on a relatively large dataset with complete scoliotic spine anatomy.

**Figure 4.**
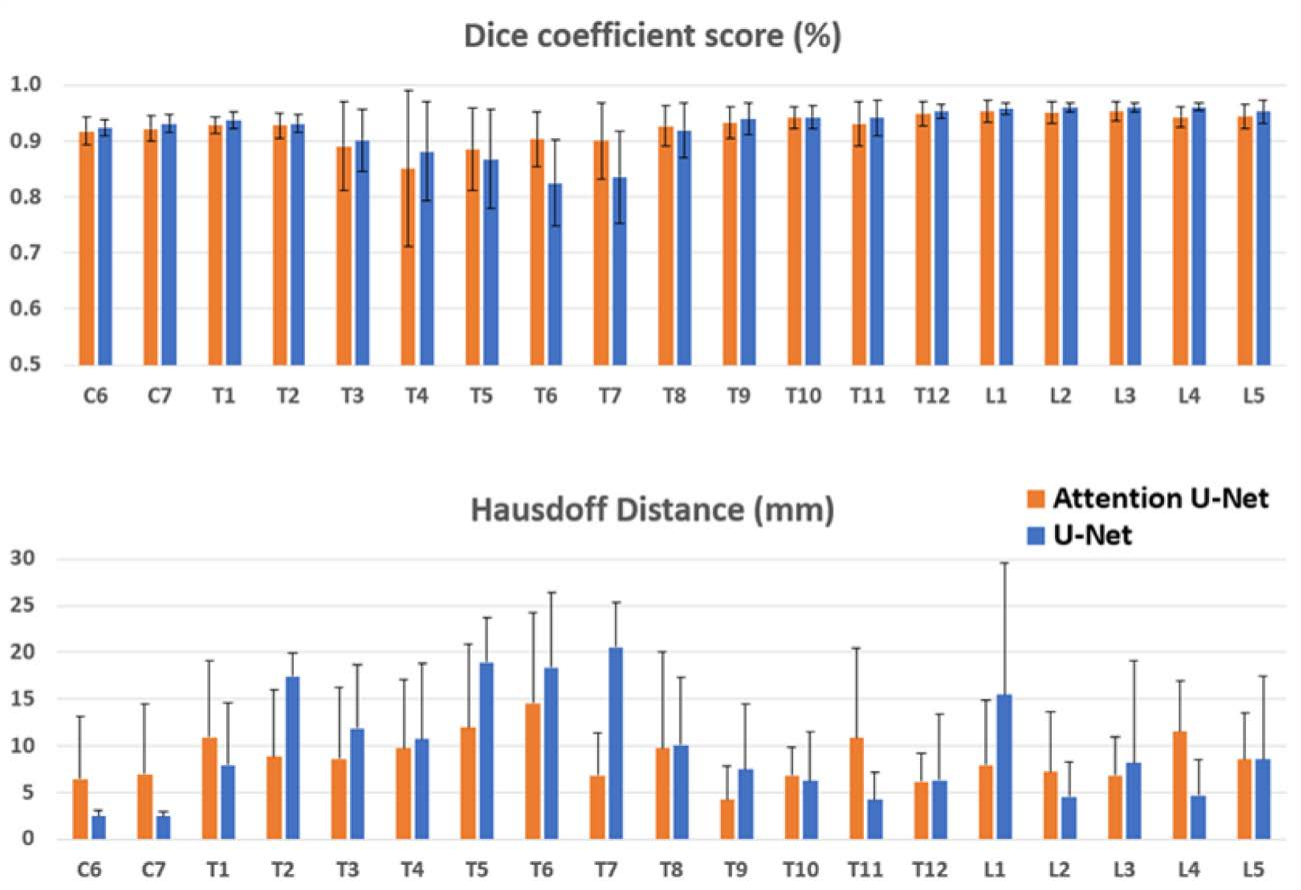
Comparison of the segmentation performance metrics (Dice Coefficient Score and Hausdoff distance) between U-Net and Attention U-Net separated by individual vertebral levels.

Furthermore, between the 2 networks employed in this study, the Attention U-Net network showed better performance in regions where scoliosis mainly exists, as illustrated in representative images at mid-thoracic regions (Fig. 3), where U-Net mislabeled several vertebral bodies while Attention U-Net provided more accurate segmentation results. This can be also evidenced by the segmentation results for individual vertebral levels shown in Figure 4, where the Attention U-Net exhibited higher Dice Coefficient Score and lower Hausdoff distance in the thoracic regions. The improved results may be associated with the inclusion of the attention mechanism,^[33]^ which has shown significant success in various image-centric applications such as image segmentation, object detection and classification. Specifically, the attention mechanism enables the model to weigh the importance of features at different locations in the input image. In context of AIS spine segmentation, it may allow the model to place more importance on the areas of the spine that are most affected by the curvature associated with scoliosis.

Our study has several limitations. Firstly, the number of AIS patients, while on par with many published papers on normal spines, is relatively small. The inclusion of more patient data for training may help improve the robustness and accuracy of segmentation performance. Second, aligned with the 1st limitation, we were not able to investigate the performance for all types of AIS due to the relatively small dataset.^[34]^ Lastly, only 2 deep learning networks were evaluated. Given the wide range of available deep learning algorithms, and the exploratory nature of our study, we only employed two of the most popular neural network architectures (U-Net family). With the hope that more data will become available for this task, future studies can aim for a more comprehensive comparative analysis of state-of-the-art deep learning algorithms.

## 5. Conclusion

In conclusion, this study successfully employed the Attention U-Net deep learning algorithm for the task of automated vertebrae segmentation using 3D CT images from AIS patients. The performance achieved is comparable to those recorded in normal spines using state-of-the-art segmentation algorithms.

##  Author contributions

**Conceptualization:** Yong Ji, Yun Peng.

**Data curation:** Yong Ji.

**Funding acquisition:** Yuliang Ma.

**Investigation:** Yuliang Ma.

**Methodology:** Xiajin Mei.

**Project administration:** Rong Tan.

**Software:** Xiajin Mei.

**Supervision:** Yuliang Ma.

**Validation:** Wenxin Zhang.

**Visualization:** Wenxin Zhang.

**Writing – original draft:** Yun Peng.

**Writing – review & editing:** Yingchun Zhang.
